# Enhancing outcome prediction of concurrent chemoradiation treatment in patients with locally advanced cervical cancer through plasma extracellular vesicle proteomics

**DOI:** 10.1016/j.heliyon.2024.e36374

**Published:** 2024-08-22

**Authors:** K. Leetanaporn, W. Chiangjong, S. Roytrakul, P. Molika, N. Janmunee, T. Atjimakul, J. Hanprasertpong, R. Navakanitworakul

**Affiliations:** a Department of Biomedical Sciences and Biomedical Engineering, Faculty of Medicine, Prince of Songkla University, Thailand; b Translational Medicine Research Center, Faculty of Medicine, Prince of Songkla University, Songkhla, Thailand; c Pediatric Translational Research Unit, Department of Pediatrics, Faculty of Medicine Ramathibodi Hospital, Mahidol University Thailand; d Functional Proteomics Technology Laboratory, National Center for Genetic Engineering and Biotechnology (BIOTEC), National Science and Technology Development Agency Thailand; e Department of Radiology, Faculty of Medicine, Prince of Songkla University, Hat Yai, Songkhla, Thailand; f Department of Obstetrics and Gynecology, Faculty of Medicine, Prince of Songkla University, Songkhla 90110, Thailand; g Department of Research and Medical Innovation, Faculty of Medicine Vajira Hospital, Navamindradhiraj University Thailand

**Keywords:** Extracellular vesicles, Ultracentrifugation, Cervical cancer, Proteomics, Treatment outcome

## Abstract

Most patients with locally advanced cervical cancer (LACC) are primarily treated using concurrent chemoradiation (CCRT); however, LACC lacks reliable predictive biomarkers. Extracellular vesicles (EVs) could define the dynamic biological response to CCRT. However, the relationship between EVs and the therapeutic response to LACC is unestablished. Thus, we aimed to determine the relationship of plasma EVs pre- and post-CCRT in 62 patients with LACC. For proteomic analyses, EVs were isolated using ultracentrifugation (UC) with size exclusion chromatography or UC alone. We found that plasma particle concentration was significantly increased post-treatment in non-responders. After CCRT, there was a decrease in proteins related to serine protease and fibrinogen, which contribute to tumor microenvironment alteration. This reduction also extended to proteins involved in innate immune and viral immune responses, correlating with reduced tumor burden. Sparse partial least squares discriminant analysis revealed 8, 13, and 19 proteins at diagnosis, one month, and three months, respectively, influencing the CCRT response. Among these, FIBG, TFR1, HBA, and FINC are prognostic markers according to The Cancer Genome Atlas tissue gene expression database. Our discriminant model demonstrated excellent specificity and negative predictive value, underscoring the model's reliability in determining responsiveness to CCRT and highlighting the potential clinical applicability of EVs in improving outcomes in LACC.

## Graphical abstract

**Figure:**
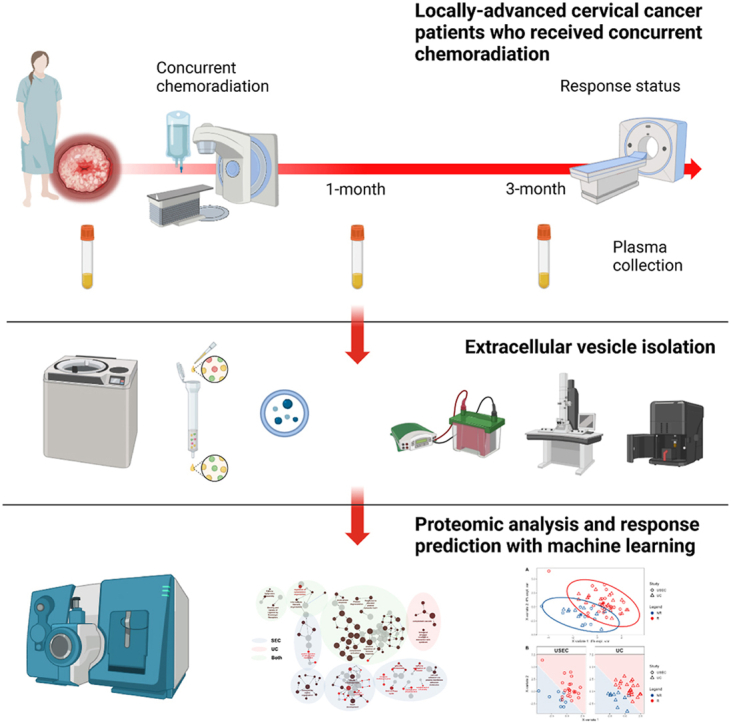

## 1 Introduction

Cervical cancer (CC) is the fourth most prevalent cancer globally and a major cause of cancer in females [1]. Although CC screening programs and vaccinations have significantly reduced its incidence in developed countries [2], its burden remains high in developing countries due to limited access to interventions available to the majority of the population [3]. In Thailand, CC remains the second most prevalent cancer in females, with 63.1 % of the patients being diagnosed at a locally advanced stage [4]. According to the Federation of Gynecology and Obstetrics (FIGO) 2018 staging system, locally advanced cervical cancer (LACC), is defined as CC stage IB3-IVA, in which the tumor has invaded beyond the operable state [5]. Thus, these groups of patients require a combination of radiation and chemotherapy as the main modality of treatment, known as concurrent chemoradiation (CCRT). Currently, the complete response rate of CCRT is 60–70 % [6], indicating that 30–40 % of patients with LACC do not completely benefit from CCRT and thus require post-therapy adjuvant treatment, which is less effective than modifying or adding modalities since the treatment initiation. Therefore, to predict CCRT response, various methods are being evaluated clinically, such as clinicopathological characteristics [7], biomarkers such as squamous cell antigen [8], and Human Papilloma Virus-DNA testing [9]. However, none of these prognostic factors reliably predict the response to CCRT before the initial treatment [[7], [8], [9]]. Moreover, identifying a group of CCRT failures using these measures does not provide additional insight into alternative therapies. Thus, it is crucial to investigate more appropriate biomarkers in order to identify CCRT non-responders, leading to efficient treatment.

Currently, the search for oncological biomarkers is focused on liquid biopsy methods such as circulating tumor cells (CTC), cell-free DNA/RNA, and extracellular vesicles (EVs) owing to their noninvasiveness, reflection of active tumor status, and ability to provide insights for precision medicine [10]. Among these, CTC, cDNA, and cRNA have limitations such as low detection and susceptibility to enzymatic degradation [11]. However, EVs can overcome these limitations. They are small, complex vesicles released from cells and play a role in intercellular communication [10]. EVs contain several types of cargo, such as proteins, DNA, RNA, and lipids, reflecting the physiological or pathological conditions of cells, which are protected from degradation by their phospholipid bilayer [12]. EVs play a role in the tumor microenvironment as protagonists of tumor growth and metastasis by transporting biological information and growth factors that promote favorable tumor microenvironments and activate pre-metastatic niches [12,13]. Clinically, EVs are enriched in most body fluids, such as blood, urine, lung fluid, semen, and saliva [12]. EV concentrations and cargo differ between patients with cancer and healthy individuals for various cancers, such as pancreatic, ovarian, lung, colorectal, and bladder cancer, along with melanoma [14], suggesting their potential as diagnostic, predictive, and treatment-monitoring biomarkers.

Determining EV concentrations or cargo can help predict therapeutic response and failure in various cancers [15]. For instance, the plasma EV concentration of glioblastoma decreases significantly after surgical resection [16]. An increased EV level in patients with breast cancer before neoadjuvant chemotherapy is associated with treatment failure, as determined using radiologic criteria [17]. The expression of EV miRNAs in post-radiation prostate cancer patients indicates a therapeutic response [18]. Previous studies on CC cells have demonstrated the potential link between EVs and cancer angiogenesis, growth, and metastasis [[19], [20], [21]]. However, limited studies have demonstrated the role of CC EVs in clinical settings. For instance, the miRNA and lncRNA expression in the EVs of vaginal lavage is significantly dysregulated in patients with CC compared to that in healthy participants [22,23]. In addition, the plasma Wnt-EV protein is a known prognostic biomarker in early-stage CC [20]. Lastly, RI3, COX5A, and SGSM3 were found to be uniquely present in the serum of CC patients compared to healthy subjects [24]. These data suggest a potential relationship between EVs and CC. However, to our knowledge, no recent publications have evaluated the relationship between EVs and the therapeutic response to LACC.

Therefore, this study aimed to determine the qualitative and quantitative relationship of plasma EVs pre- and post-CCRT in patients with LACC to identify its potential as a promising biomarker for predicting treatment response and navigating future treatment plans.

## 2 Materials and methods

### 2.1 Patient recruitment and data collection

This prospective cohort study involved patients newly diagnosed with LACC at Songklanagarind Hospital. The inclusion criteria were patients with CC stage IIB-IVA according to International Federation of Gynecology and Obstetrics (FIGO) 2018 [5] who received CCRT as the primary treatment, age 18–60 years, histopathological diagnosis of squamous cell carcinoma, adenocarcinoma, or adenosquamous carcinoma. The exclusion criteria were as follows: suspected distant cancer metastasis on computed tomography (CT) or clinical examination, synchronous or metachronous cancer, human immunodeficiency virus infection, pregnancy, or allergy to CT contrast.

The following information on clinicopathological characteristics was obtained: age, body mass index, stage according to FIGO 2018, histopathology, and tumor size. Biochemical data were collected at the first diagnosis, including hemoglobin, white blood cell count, platelet count, and creatinine levels. This study was approved by the Human Research Ethics Unit, all patients in the study provided written consent before participation (REC-63-465-4-2).

### 2.2 Method details

#### 2.2.1 Concurrent chemoradiation protocol

Patients with LACC received CCRT according to the standard protocol of Songklanagarind Hospital, which aligns with the National Comprehensive Cancer Network (NCCN) guideline [5]. The radiation regimen involved external beam radiation therapy (EBRT), involving conventional (2D) and 3D conformal radiotherapy, occasionally including intensity-modulated radiation therapy. Briefly, a 45–50 Gy dose of whole-pelvis EBRT was delivered in 1.8–2 Gy daily fractions, five days per week, with considered boost to the lymph nodes, parametrium, and pelvic sidewalls, totaling 56–60 Gy. Simultaneously, platinum-based chemotherapy (cisplatin 40 mg/m^2^ or carboplatin AUC 2) was administered weekly during EBRT. Brachytherapy was administered as a high-dose regimen. Following treatment completion, patients were monitored according to a predetermined follow-up protocol. The patients’ response status was determined following the NCCN guideline [5] at three months post-treatment using clinical information and CT data, according to the RECIST criteria [45]. Status was defined as a response for no evidence of cancer by clinical examination and CT after three months of CCRT, and non-response for the presence of residual tumor or fresh lesions by clinical examination or CT after three months of CCRT.

#### 2.2.2 Plasma preparation

Blood was drawn at diagnosis, one month-post treatment, and three months-post-treatment. Further, 10 mL blood was centrifuged at 2500×*g* at 4 °C for 15 min twice, to eliminate debris, red blood cells, white blood cells, and platelets. The platelet-free plasma was stored at −80 °C until use.

#### 2.2.3 Nanoparticle tracking analysis

The size and concentration of the plasma particles were measured using NTA via Nanosight NS300 (Malvern Panalytical, Malvern, UK). After thawing, each sample was diluted 1:500–1:1000 and recorded at 25 °C, 30 s/video five times for two replicates per specimen. The NTA software determined particle size distribution and concentration (Malvern Panalytical, Malvern, UK).

#### 2.2.4 EV isolation using ultracentrifugation

After thawing at 37 °C for 5 min, plasma was serially centrifuged at 4 °C at the following speeds: 2500×*g* for 15 min to remove residual debris, 17,000–20,000×*g* for 45 min to eliminate cell fragments and large particles, and 120,000×*g* for 90 min to obtain EV pellets. The sediments were resuspended in phosphate-buffered saline (PBS) and washed using ultracentrifugation (UC) at 120,000×*g* for 90 min. The pellets were resuspended again in 40 μL PBS. For subsequent size exclusion chromatography (SEC), the pellets were resuspended in 500 μL PBS for protein digestion.

#### 2.2.5 EV purification using SEC

After UC, plasma EVs were purified using qEV column gen 1 (Izon Science, Christchurch, New Zealand). According to the user's manual, 500 μL of PBS-suspended EVs was loaded onto qEV columns within a temperature of 18–24 °C. The eluted fractions 1–4 were collected using an automatic fraction collector (Izon Science, Christchurch, New Zealand) and centrifuged at 120,000×*g* for 90 min to obtain purified EVs. The pellets were resuspended and pooled in 40 μL PBS for protein digestion.

#### 2.2.6 EV characterization

##### 2.2.6.1 Transmission electron microscopy (TEM)

The negative fixation method was used for TEM visualization. EVs were fixed with 2.5 % glutaraldehyde in PBS for 30 min; 10 μL of each EV sample was then placed on a Formvar-coated carbon grid and incubated for 30 min. Thereafter, 10 μL of 2 % uranyl acetate was added on the grid for negative staining and incubated in a dark room for 10 min. The excess solution was removed by blotting the grid at a 45-degree angle. The grid was visualized using a JEM-2010 TEM (JEOL, Tokyo, Japan) with 25,000× magnification at 80 kV at the Scientific Equipment Center of Prince of Songkla University.

#### 2.2.7 Western blotting

Protein concentration was measured using the Bradford assay with bovine serum albumin as a standard reference. Each 20 μg of protein was separated using an 8–10 % sodium dodecyl sulfate-polyacrylamide gel electrophoresis at 120 V for 120 min. The separated proteins were transferred onto a polyvinylidene fluoride membrane at 100 V for 120 min and blocked with 5 % non-fat milk in 1 % Tris-buffered saline with Tween 20 (TBST) at room temperature for 60 min. The membrane was incubated in 1 % TBST at 4 °C overnight with the following diluted primary antibodies: rabbit monoclonal antibodies (mAb), anti-CD9 (1:500), rabbit mAb, anti-CD63 (1:500), mouse mAb anti-Alix (1:500), mouse mAb anti-cytochrome C (1:500), and anti-albumin (1:1000). The membranes were then incubated with anti-rabbit horseradish peroxidase (HRP)-conjugated (1:1000) or anti-mouse HRP-conjugated (1:1000) secondary antibody at room temperature for 60 min. Finally, an anti-rabbit IgG-HRP-linked antibody (1:1000) was used to probe the immunoglobulin (Ig)G proteins. The membrane was washed thrice with TBST for 10 min each. The proteins were visualized using enhanced chemiluminescence (GE Healthcare Bioscience, PA, USA).

#### 2.2.8 Bottom-up EV proteomic study

##### 2.2.8.1 Protein digestion

Proteins were extracted from EVs using sodium dodecyl sulfate in the presence of a reducing agent. Subsequently, 20 μg proteins were reduced with dithiothreitol (Sigma Aldrich, St. Louis, MO, USA) and alkylated in the dark using 2-iodoacetamide (Sigma Aldrich). The alkylated proteins were digested for 20 h with 1:50 trypsin. After arresting the reaction with 0.1 % formic acid, the peptides were clarified using a C18 solid phase extraction disk and resuspended at 1 μg/μL in 0.1 % formic acid.

##### 2.2.8.2 Proteomics analysis using liquid chromatography–tandem mass spectrometry

The clarified peptides (1 μg) were subjected to liquid chromatography-tandem mass spectrometry analysis. In the UC cohort, the digested peptides were injected on an UltiMate 3000 Nano/Capillary LC System (Thermo Scientific) coupled with a μ-precolumn (C18 PepMap 100; Thermo Scientific) and an Acclaim PepMap RSLC analytical column (75 μm × 15 cm, C18, nanofiber; Thermo Scientific) at 60 °C. The elution gradient comprised of 5–55 % solvent B (0.1 % formic acid in 80 % acetonitrile) over 30 min. MS and MS/MS spectra were acquired in a data-dependent (DDA) manner using an Impact II mass spectrometer (Bruker Daltonics, Billerica, MA, USA) operated in positive ion mode over a mass range (*m*/*z*) of 300–1800. In the UC with SEC (USEC) cohort, peptides were injected into an ultra-high-performance nanoflow LC system (Eksigent, Dublin, CA, USA) equipped with a C18 trap column (Nano Trap TP-1, 3 μm 120 Å, 10 mm × 0.075 mm and bioZen Peptide Polar C18) and analytic column (75 μm × 15 cm, C18 particle sizes of 3 μm, 120 Å; Phenomenex, CA, USA). Gradient elution was performed using a concentration of 3–40 % solvent B, spanning over 70 min. Spectral data were acquired using a TripleTOF 6600+ instrument (AB Sciex, Toronto, Canada) operated in positive ion mode in a DDA manner for spectral library generation and in a data-independent (DIA) with SWATH acquisition at mass window of 7 *m*/*z* and 1 *m*/*z* overlapping windows were used for ion identification and quantification.

### 2.3 Quantification and statistical analysis

#### 2.3.1 Spectral data processing

All files were converted into “. mzml” using MSConvert (Proteowizard) with the “peakPicking 1-” option before processing with Fragpipe 21.0 [46]. The spectral data were searched against the UniProt Human proteome database (UP000005640; 11/2023) with precursor and fragment tolerances of 20 and 30 ppm, respectively, trypsin (KR cut) enzymatic semi-digestion, carbamidomethyl of C-fixed modification, oxidation for M variable modification, charge stage 0/1/2/3, and allowed up to two missed cleavages. Ion intensities were quantified and normalized at the precursor level for both unique and razor peptides with a match between runs. DIA data were identified from the DDA spectral library and DIA signal extraction, then quantified and normalized with a match between runs using DIA-NN [47]. The precursor, peptide, and protein identification were validated at 1 %, 1 % and 5 % FDR.

#### 2.3.2 Clinicopathologic characteristics

Clinicopathological data and particle concentrations are expressed as percentages, means, standard deviations, and ranges. Fisher's exact test was used for proportion tests. The means and medians of the two samples were compared using *t*-test/Wilcoxon rank sum tests. The means and medians of three or more samples were compared using analysis of variance/Kruskal–Wallis test. Statistical significance was set at p ≤ 0.05.

#### 2.3.3 Protein expression data processing

The protein intensity data were analyzed using the R program 4.3.1. Any missing data were imputed through random forest imputation using the R missForest package. The data were then transformed into log2 values. The data were further preprocessed by z-score normalization if integration of the USEC and UC cohorts was required. The intensity was normalized to 0–1 range for visualization.

#### 2.3.4 Pathway analysis

Protein pathway annotation was performed using the Gene Ontology and Reactome databases. The annotated pathways were used to perform overexpression analysis and network visualization. The significance threshold of significant pathways was determined using an exact test under hypergeometric distribution at a p-value of ≤0.05, with false discovery being adjusted using the Benjamini–Hochberg method. The semantic similarity between significant pathways was determined using the Wang method. Pathways with minimum similarity >0.5 were clustered using the Jaccard similarity. All data analyses were performed using R clusterProfiler package and Cytoscape software.

#### 2.3.5 Clinical response prediction from protein data

The protein intensity and clinical response data were used to construct a model for prediction. Data protein intensity was preprocessed with z-score normalization. The clinical response variables were used as categorized data. The “mixOmics” package used the supervised Partial Least Squares Discriminant Analysis (sPLS-DA) to integrate data from both cohorts for feature selection using the Mahalanobis distant and balanced error rate metrics, with the leave-one-out cross-validation. The final prediction model was performed separately in the USEC cohort as the training set and validated using the UC cohort as the test set.

## 3 Results

### 3.1 Patient characteristics

A total of 73 patients were eligible for the study. Among these, 6 cases were excluded for referral to other hospitals, 3 cases for loss follow-up, and 2 cases for denied participation. Of the 62 patients who remained eligible in this study, 44 patients were CCRT responders, and 18 were CCRT non-responders. The median patient age was 50 (Q1–Q3; 41–56 years). The evaluated characteristics did not differ significantly between responders and non-responders, except for the FIGO stage, wherein stages 2B or lower had a higher proportion of responding patients than those in the other groups (2B or lower vs 3B, 86 % vs. 50 %, p = 0.04; 2B or lower vs. 3C or higher, p = 0.037). The cohort was divided into the discovery (n = 31) and verification (n = 31) groups. All the clinicopathological characteristics are presented in Table 1.

**Table 1:** Clinicopathologic characteristics of locally advanced cervical cancer in the cohort.

Characteristic	Response, N = 44^a^	Non-Response, N = 18^a^	p-value^b^
**Age (year)**	50 (42, 56)	46 (39, 54)	0.3
**BMI (kg/m^2^)**			>0.9
< 18.5	2 (67 %)	1 (33 %)	
18.5–24.9	19 (70 %)	8 (30 %)	
≥ 25	23 (72 %)	9 (28 %)	
**FIGO Stage**^c^			**0.046**
2B or lower	24 (86 %)	4 (14 %)	
3B*	4 (50 %)	4 (50 %)	
3C or higher*	16 (62 %)	10 (38 %)	
**Histology**^d^			0.5
SCC	35 (71 %)	14 (29 %)	
ADC	6 (60 %)	4 (40 %)	
ASC	3 (100 %)	0 (0 %)	
**Tumor size (cm)**			0.2
<4	11 (92 %)	1 (8.3 %)	
≥4	33 (66 %)	17 (34 %)	
**Hemoglobin (g/dL)**			0.3
< 10	6 (55 %)	5 (45 %)	
≥ 10	38 (75 %)	13 (25 %)	
**WBC count (cells/mm^2^)**^d^			0.7
< 10000	29 (73 %)	11 (28 %)	
≥ 10000	15 (68 %)	7 (32 %)	
**Platelets (cell/mm^2^)**			0.3
< 450000	8 (57 %)	6 (43 %)	
≥ 450000	36 (75 %)	12 (25 %)	
**Creatinine (mg/dL)**	0.63 (0.57, 0.72)	0.67 (0.60, 0.73)	0.5
**Cohort**^d^			0.6
USEC	23 (74 %)	8 (26 %)	
UC	21 (68 %)	10 (32 %)	

a Median (IQR); n (%).

b Wilcoxon rank sum test; Fisher's exact test; Pearson's Chi-squared test.

c * A pair that was not significantly different.

d SCC = Squamous cell carcinoma, ADC = adenocarcinoma, ASC = adenosquamous carcinoma, WBC = white blood cell, USEC = ultracentrifugation with size exclusion chromatography, UC = ultracentrifugation.

### 3.2 Temporal changes in plasma particle concentration

**Fig. 1:**
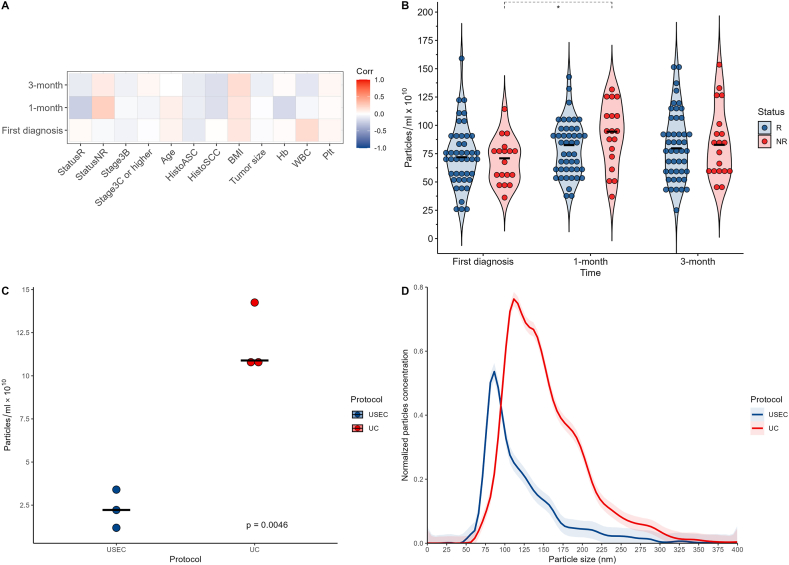
Heatmap of plasma particles and clinicopathologic characteristics (A). Particle concentrations in the response and non-response groups at first diagnosis, one-and three-month after CCRT treatment (B). Extracellular vesicle isolation yield (C) and Particle size distribution using each protocol (D). Asterisks denote statistical significance (*: p ≤ 0.05). **Abbreviations:** R = responder, NR = non-responder, ASC = adenosquamous carcinoma, SCC = squamous cell carcinoma, BMI = body mass index, Hb = hemoglobin, Plt = platelets, UC = ultracentrifugation; USEC = ultracentrifugation followed by size exclusion chromatography.

The overall median concentration of particles was 7.65 × 10^11^ particles/mL (Q1–Q3 5.76 × 10^11^–9.47 × 10^11^ particles/mL). Pearson's correlation analysis showed no significant correlation between particle concentrations and clinicopathological characteristics at any time point (Fig. 1A). However, the particle concentration of non-responder significantly increased at one-month post-treatment compared to the first diagnosis (mean concentration 9.14 × 10^11^ vs. 6.87 × 10^11^ particles/ml, p = 0.048, Fig. 1B).

### 3.3 EV concentration and particle distribution

To establish an EV isolation protocol, plasma from three excluded patients (n = 6) was used to pilot the isolation procedures. After removing debris and large EV particles, the supernatants were subjected to UC only or USEC. NTA revealed that USEC yielded less particle concentration than UC (mean concentration 2.26 × 10^10^ vs. 1.19 × 10^11^ particles/mL, p = 0.046) (Fig. 1C). Moreover, the particle range from USEC was smaller and narrower than that from UC (median size 85.5 nm [Q1–Q3, 104.5–140.5 nm] and 142.5 nm [Q1–Q3, 104.5–180.5 nm], respectively, Fig. 1D).

### 3.4 EV characteristics and morphology

**Fig. 2:**
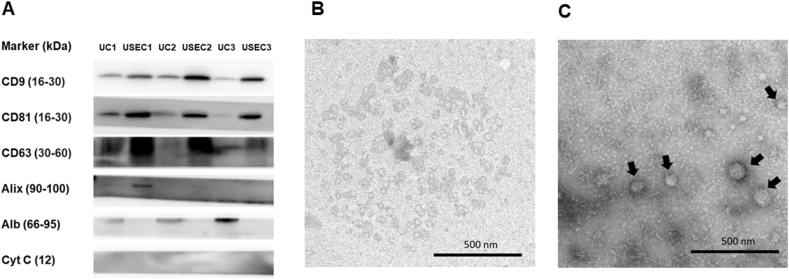
Western blotting analysis of EV markers using UC and USEC protocol (A). Representative transmission electron microscopy image of EVs isolated from the USEC (B), and UC protocol (C) with 25,000× magnification. **Abbreviations:** kDa = kilodaltons, Alb = albumin, Cyt C = cytochrome C, UC = ultracentrifugation, USEC = ultracentrifugation followed by size exclusion chromatography, nm = nanometers.

Consequently, western blotting was performed to confirm the presence of EV markers and assess the purity of EV isolates from both protocols (Fig. 2A, Supplementary Fig. 1). The findings revealed the presence of transmembrane proteins (CD9, CD81, and CD63) using both protocols and the absence of a negative marker (Cytochrome C), confirming the elimination of the cellular compartment. However, cytosolic proteins (such as Alix) were present in only one sample. Moreover, the additional SEC purification resulted in a higher concentration of EV markers and lower albumin contamination in the isolates than those from UC alone.

The isolates were further subjected to morphological assessment using TEM (Fig. 2B and C). Images from both methods showed multiple circular-shaped particles with a lipid bilayer morphology. Notably, the EV pellets isolated by USEC contained a homogenous population of circular bilayer enclosed vesicles with minimal evidence of protein contamination (very dark staining material found in UC isolation method) compared to those obtained using UC.

### 3.5 EV proteomics profiles and comparisons with public EV database

**Fig. 3:**
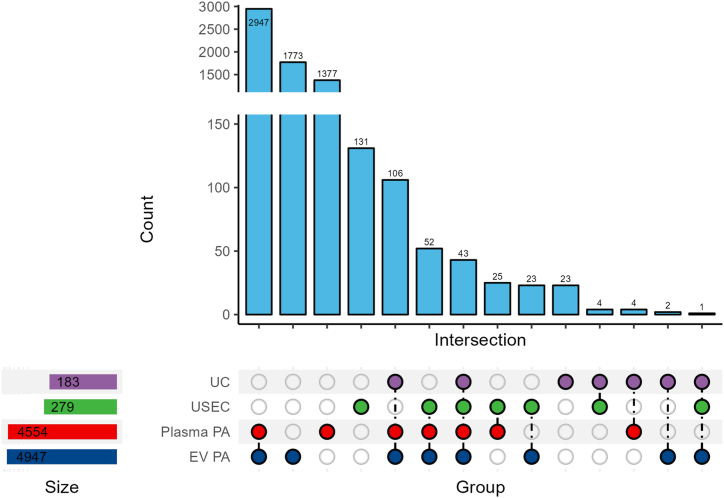
An upset plot of proteins was identified from our protocols (UC and USEC) and public databases. Each column indicates the number of proteins that were present in the combination of the mentioned cohorts (e.g., Plasma PA + EV PA = 2947 proteins, UC + plasma PA + EV PA = 106 proteins, UC + USEC + Plasma PA + EV PA = 43 proteins). **Abbreviations:** UC = ultracentrifugation, USEC = ultracentrifugation followed by size-exclusion chromatography, EV = extracellular vesicle, PA = Peptide Atlas Database.

Before cohort separation, we analyzed the general protein profile from each protocol. In total, 433 unique proteins were identified in both UC and USEC cohorts (Fig. 3). Among these, 279 and 183 proteins were identified in the USEC and UC cohorts, respectively. Comparison of these results with the PeptideAtlas database, which is the curated database reanalyzed from previously published proteomics from neat and EV plasma [25]. The comparative data demonstrated substantial overlap with publicly available data (USEC 51.61 % [144/279] and UC 85.24 % [156/183]), with 43 unique proteins identified in all groups. Additionally, USEC identified more EV-specific proteins that were not present in the plasma (8.24 % [23/279] vs. 1.09 % [2/183] in UC). However, USEC also identified more plasma proteins not found in the EV database than that with UC (8.96 % [25/279] vs. 2.2 % [4/183], respectively). No protein could be simultaneously identified in our experiments and plasma; however, not in the EV database. Protein intensities revealed that the unique proteins recovered using UC were less abundant. However, the distribution of protein abundance using USEC was more generalized (Supplementary Fig. 2).

### 3.6 Different protein pathways were enriched between USEC and UC enrichment

**Fig. 4:**
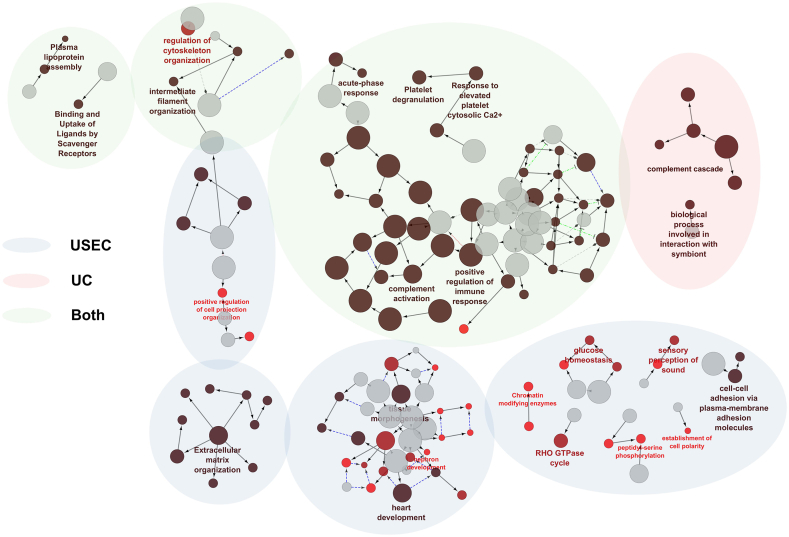
Cytoscape network result of proteins enriched in each protocol. **Blue:** proteins uniquely enriched by USEC, **Red:** proteins uniquely enriched by UC, **Green:** proteins enriched by both methods. **Abbreviations:** UC = ultracentrifugation, USEC = ultracentrifugation followed by size-exclusion chromatography. (For interpretation of the references to color in this figure legend, the reader is referred to the Web version of this article.)

To further elucidate the differences in protein representation between the USEC and UC methods, the proteins found in each method were subjected to an overrepresentation analysis (ORA) of the gene ontology and Reactome pathway, irrespective of their presence in previously discovered databases (231, 138, and 48 proteins for USEC, UC, and both cohorts, respectively; Fig. 4). Only terms containing more than eight proteins were included in the ORA to increase pathway term specificity. Cytoscape results demonstrated that the majority of the proteins in the USEC cohort (blue) were related to cell organization, extracellular matrix reorganization, and organ development, with minor terms related to cell communication, enzymes, and hemostasis. In contrast, protein terms from UC (red) primarily represented complement cascade activation. Finally, the proteins present in both cohorts (green) were related to immune response regulation, complement activation, lipoprotein assembly, and cell filament reorganization. Thus, most of the protein terms captured by UC were also present in USEC. Moreover, the USEC method also identified diverse functional proteins.

### 3.7 Dynamic protein expression profile of LACC patients with CCRT over time

**Fig. 5:**
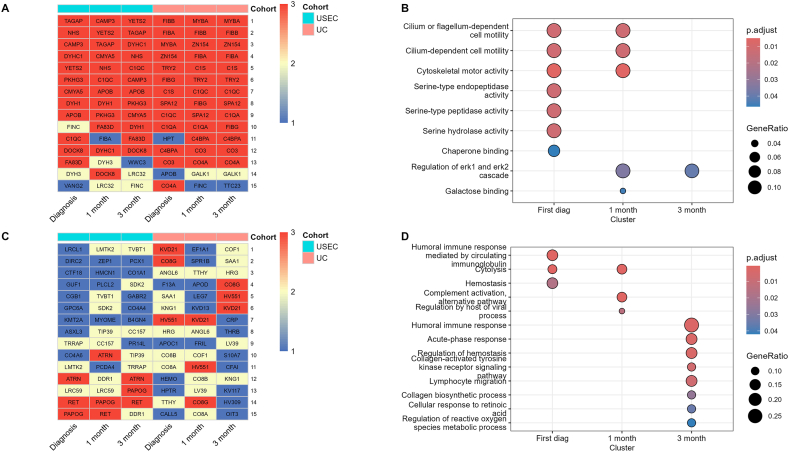
Top 15 abundant proteins according to time point from each protocol (A). Dot plot of significant pathways enriched from top 15 proteins in each time point (B). Last 15 abundant proteins according to time point from each protocol (C). Dot plot of significant pathways enriched from the last 15 proteins in each time point (D). The protein list from each cohort found at 1, 2, and 3 time points are indicated in blue, yellow, and red, respectively. **Abbreviations:** UC = ultracentrifugation, USEC = ultracentrifugation followed by size-exclusion chromatography. (For interpretation of the references to color in this figure legend, the reader is referred to the Web version of this article.)

To observe the overall change in protein expression throughout the treatment period, we compared the mean intensity of each protein across all time points in both UC and USEC cohorts (Supplementary Table 1) and extracted the top and bottom 15 most abundant proteins for visualization and pathway analysis (Fig. 5). The heatmaps of all proteins contributing to each pathway are presented in the supplementary files. For the top 15 abundant proteins, we observed that 83.3 % were presented across all time points. Pathway analysis of these proteins revealed that most proteins were associated with serine peptidase activity and cilium signaling at the time of initial diagnosis. However, at one- and three-months post-treatment, the presence of serine peptidase proteins diminished, while proteins related to the regulation of the ERK pathway were prominent at a later time point. We observed that the protein level was decreased compared to that at the initial diagnosis (Supplementary Fig. 2), but was more prominent in the pathway enrichment due to a decrease in the other proteins mentioned earlier.

However, the presence of the 15 least abundant proteins was variable, with only 20 % consistently appearing across all time points. Pathway analysis revealed that these proteins were related to the humoral immune response. Additionally, the impaired hemostasis mechanism observed at the initial diagnosis returned to higher baseline activity after three months, disappearing from overrepresentation pathways. Moreover, a shift away from the innate immune response was observed from decreased acute-phase response and lymphocyte migration activity-related proteins. Notably, proteins related to the regulation of viral immune processes were less present at one-month post-treatment but not at three months. However, protein intensity data revealed that the expression of proteins was decreased but was obscured by a more significant decrease in other proteins (Supplementary Fig. 2).

### 3.8 Constructing a model to differentiate the response status of LACC patients with CCRT

**Fig. 6:**
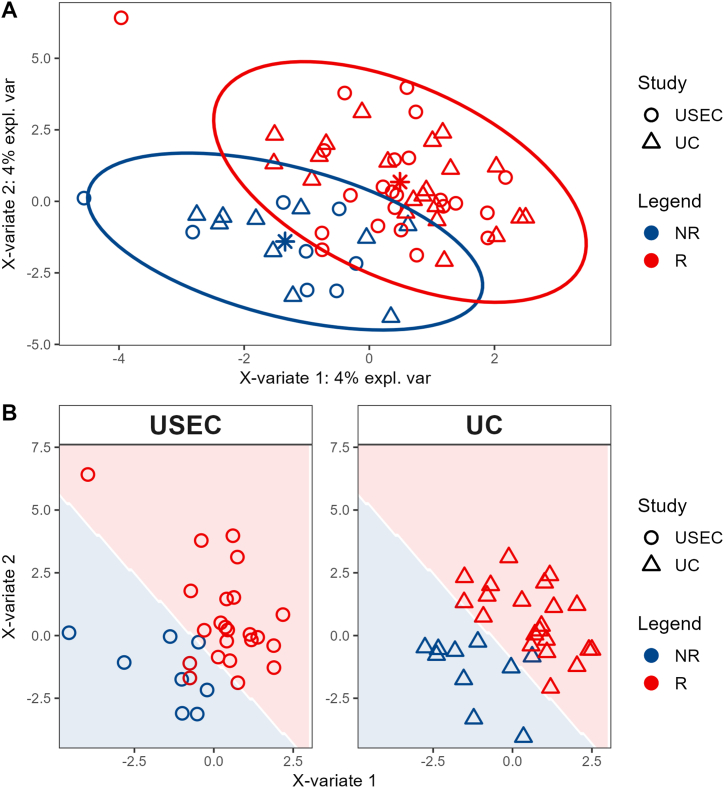
Supervised Partial Least Squares Discriminant component analysis of the combined cohort (A) and individual cohort (B). **Abbreviations:** UC = ultracentrifugation, USEC = ultracentrifugation followed by size-exclusion chromatography, NR = non-responder, R = responder.

We employed sPLS-DA with leave-one-out cross-validation to identify potential proteins that could discriminate patients' CCRT responses. The analysis included 48 unique proteins that were found in both USEC (23 responders and eight non-responders) and UC cohorts (21 responders and 10 non-responders) (Table 1, Fig. 3), as these proteins were consistently present regardless of the isolation procedure. Firstly, we employed a multivariate sPLS-DA model to determine significant proteins from a combination of both cohorts. The multivariate model demonstrated a suitable separation between non-responders and responders, irrespective of the isolation procedure (Fig. 6).

This model identified eight, 13, and 19 proteins at diagnosis, one month, and three months, respectively, influencing the CCRT response. Among these, four proteins (IGLL5, CO3, IGJ, and APOC4) showed conflicting response odd directions among time points (e.g., increased non-response odds at diagnosis but decreased odds at one month) and were not considered as potential variables. Among the remaining 29 proteins examined, nine proteins (IGKC, FIBA, FIBB, FIBG, HV315, C1QB, APOB, HBB, and SIG16) that exhibited increased abundance correlated with a lower likelihood of response (Table 2).

**Table 2:** Coefficients of important proteins according to the sPLS-DA model.

ID	Protein	Genes	Coefficient of response		
			Diagnosis	1 month	3 months
P01834	IGKC	*IGKC*	−0.14	–	–
P02654	APOC1	*APOC1*	0.3	–	–
**P02679**	**FIBG**	***FGG PRO2061***	**−0.07**	**-**	**-**
P04908	H2A1B	*H2AC4 H2AFM HIST1H2AB; H2AC8 H2AFA HIST1H2AE*	0.27	–	–
P10909	CLUS	*CLU APOJ CLI KUB1 AAG4*	0.33	–	0.2
P60709	ACTB	*ACTB*	0.8	–	–
**P69905**	**HBA**	***HBA1; HBA2***	**0.03**	**-**	**-**
A0A075B6H7	KV37	*IGKV3-7*	–	0.05	0.33
A0A0A0MRZ8	KVD11	*IGKV3D-11*	–	0.29	0.15
A0A0B4J1V0	HV315	*IGHV3-15*	–	−0.11	−0.03
O43866	CD5L	*CD5L API6 UNQ203/PRO229*	–	0.18	0.07
P01023	A2MG	*A2M CPAMD5 FWP007*	–	0.16	0.44
P01619	KV320	*IGKV3-20*	–	0.22	0.24
P02649	APOE	*APOE*	–	0.08	–
P02746	C1QB	*C1QB*	–	−0.32	–
P04114	APOB	*APOB*	–	−0.02	–
P0C0L4	CO4A	*C4A CO4 CPAMD2*	–	0.14	–
P68871	HBB	*HBB*	–	−0.06	–
A0A0J9YX35	HV64D	*IGHV3-64D*	–	–	0.04
A6NMB1	SIG16	*SIGLEC16 SIGLECP16*	–	–	−0.22
P01859	IGHG2	*IGHG2*	–	–	0.28
P02655	APOC2	*APOC2 APC2*	–	–	0.08
P02671	FIBA	*FGA*	–	–	−0.13
P02675	FIBB	*FGB*	–	–	−0.01
P02747	C1QC	*C1QC C1QG*	–	–	0.35
**P02751**	**FINC**	***FN1 FN***	**-**	**-**	**0.55**
**P02786**	**TFR1**	***TFRC***	**-**	**-**	**0.2**
P08519	APOA	*LPA*	–	–	−0.04
Q9UPR0	PLCL2	*PLCL2 KIAA1092 PLCE2*	–	–	0.02

* Bold labels are proteins with poor prognostic markers in The Cancer Genome Atlas cohort.

### 3.9 External validation using the CC public database

**Fig. 7:**
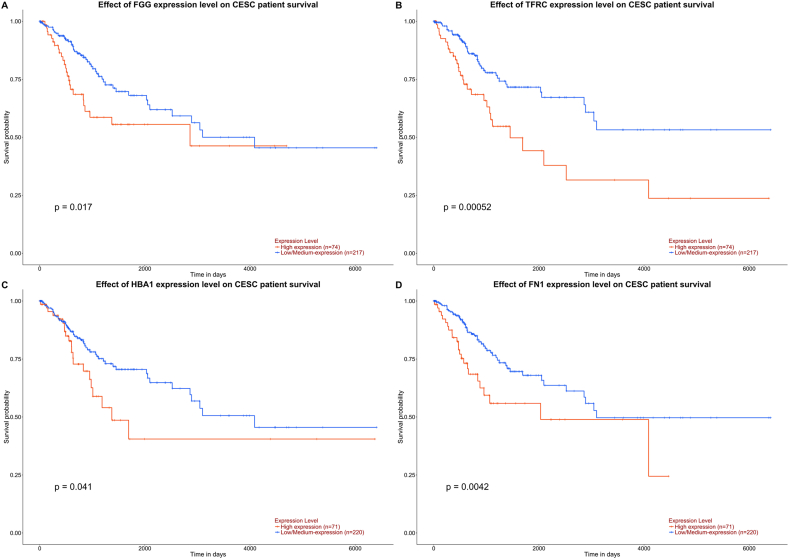
Precursor genes (mRNA) expression from The University of Alabama at Birmingham Cancer data analysis portal tissue expression that influence patient survival such as *FGG* (A), *TFRC* (B), *HBA1* (C), and *FN1* (D).

To identify potential proteins that influence clinical outcomes, we validated all potential proteins using the UALCAN public database [26], (Fig. 7), which is the portal website that extracts survival outcomes according to mRNA expression from tissues of CC patients in The Cancer Genome Atlas (TCGA) cohort. From 29 proteins, we identified four mRNAs that were precursors of our identified proteins and that had a significant influence on the survival outcomes: *FGG* (p = 0.017, FIBG), *TFRC* (p = 0.00052, TFR1), *FN1* (p = 0.0042, FINC), and *HBA1* (p = 0.041, HBA).

### 3.10 Assessment of predictive power on separate cohorts

**Fig. 8:**
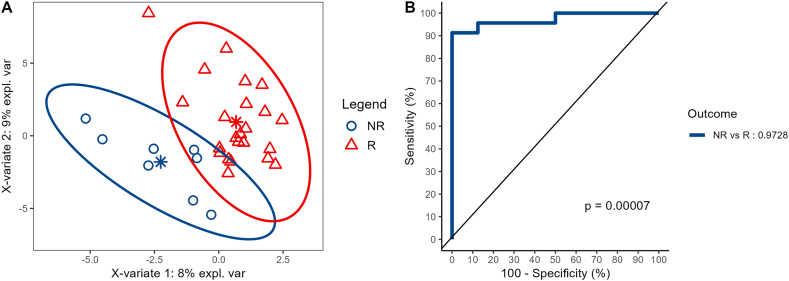
Supervised Partial Least Squares Discriminant Analysis performance on the USEC (as the discovery) cohort. **A:** Component analysis, **B:** receiver operating characteristic curve.

We analyzed the USEC and UC cohorts separately to determine the true predictive power of a unique protein set. We used the sPLS-DA model separately on USEC as the training set and validated it on the UC set (Fig. 8). The receiver operating characteristic curve of the training model showed that the area under the curve (AUC) for the training set and the verification set were 0.9783 and 0.6612, respectively. Furthermore, the individual metrics in the verification set showed modest positive prediction metrics (sensitivity = 0.10, positive predictive value = 0.50, and Kappa = 0.07) but commendable negative prediction performance (specificity = 0.95, negative predictive value = 0.69, and McNemar's p-value = 0.026, Table 3).

**Table 3:** Performance of the trained model on UC (as the verification) cohort.

	Reference	
**Prediction**	NR	R
NR	1	1
R	9	20
**Accuracy**	: 0.68 (0.49, 0.83)	
**No Information Rate**	: 0.68	
**p-value [Acc > NIR]**	: 0.58	
**Kappa**	: 0.07	
**McNemar's Test p-value**	: 0.026	
**Sensitivity**	: 0.10	
**Specificity**	: 0.95	
**Pos Pred Value**	: 0.50	
**Neg Pred Value**	: 0.69	
**“Positive” Class**	: NR	

## 4 Discussion

While treatment guidelines for CC patients have remained consistent, the response rates, particularly in advanced stages, remain unsatisfactory. Therefore, a deeper understanding of the patient response mechanisms to CCRT is essential. In this study, we characterized plasma EVs at clinically significant time points from patients undergoing CCRT to elucidate dynamic changes in the biological response of patients. Although the particle concentrations in the plasma of patients at each time point did not correlate significantly with clinicopathological characteristics, an increase in particle concentration was observed in non-responders at one-month post-treatment. This observation is consistent with that of Malla et al., who found increased serum EV concentrations in patients with prostate cancer after radiation therapy [18]. We also observed a modest increase in particle concentrations among responders, indicating an increase in the systemic response of the body in both responders and non-responders.

Regarding EV separation methods, the yield of EV particles from the UC protocol was higher than that from the USEC protocol, with a marginally different particle distribution. Western blotting results of the EV marker population revealed the presence of tetraspanin markers in our pilot samples; Alix proteins were present in only two samples. These findings highlight the importance of using multiple EV markers to validate the isolation protocol, as suggested by the standard recommendation [27]. Moreover, Alix is a common EV marker, regardless of the cell sub-population [28,29]. Our results indicate that EV variation in clinical samples may be more significant depending on human biological variations and current conditions. Differences in the EV Alix abundance in body fluids can potentially be used to diagnose and monitor certain cancers [30,31].

Regarding proteomic profiling, previous studies have explored the efficacy of each method with some variations [[32], [33], [34]]. In our study, the USEC protocol resulted in higher unique protein retrieval than the UC protocol. Additionally, the USEC method resulted in a higher percentage yield of unique EV proteins than those with the UC method, using the PeptideAtlas database. This result is consistent with that described by Wei et al. and Turner et al., who stated that a combination of methods results in better protein identification than that with either method alone, particularly when UC precedes SEC [34,35]. However, the protein number from UC outperformed that from USEC when the uniqueness from plasma was not considered, reflecting different EV population enrichments with each method. Furthermore, EV protein yield from the UC method did not exceed that from USEC with an increase in the number (up to five times) or length of the cycle [32]. Nevertheless, the analysis time of the optimized method poses a significant constraint with an increase in the number of samples.

Considering dynamic changes in proteins with time, we identified proteins related to serine protease which contribute to extracellular matrix remodeling and immune modulation in the tumor microenvironment [36]. Reduction in these proteins after CCRT demonstrated a reduction in tumor activities. Additionally, there was also a reduction, albeit less prominent, in the expression of fibrinogen proteins. These proteins mediate downstream phosphorylation in the MAPK/ERK pathway [37]. Notably, the higher level of FIBG, one of the fibrinogen proteins that contributes to the ERK pathway, was correlated with an increased probability of non-response after treatment as per our sPLS-DA model. On the other hand, the last 15 proteins showed a decrease in innate immune regulation, depicted by a reduction in humoral immune response and lymphocyte migration. Moreover, the protein-related regulation of viral immune response was also decreased, correlating with a decrease in tumor burden after treatment initiation.

In addition to serving as a resource for understanding temporal biological changes after CCRT, some proteins could discriminate between responders and non-responders, thus serving as potential clinical biomarkers. We employed a rectangular strategy for proteomics using the simultaneous proteins presented in a separate cohort to improve coverage and reduce time complexity in the clinical setting [38]. The results showed multiple proteins contributed to the response status prediction at each time point. Of these, dysregulation of four protein gene precursors, *FGG* (FIBG), *TFRC* (TFR1), *HBA1* (HBA), and *FN1* (FINC), were found to be poor prognostic markers in the TCGA cohort [26]. Transferrin receptor (*TFRC*) and fibronectin 1 (*FN1)*, significant contributors at three months, contributed to the tumor regulation of viral production and multiple tumor cellular activities in cell line models [39,40]. TFRC is involved in iron metabolism and regulates various genes related to cell death and viral replication, which contribute to cancer aggression [39]. Moreover, CC subpopulations with CD71 (transfer receptors) exhibit cancer aggressiveness and radiological resistance, which can be sensitized by CD71 suppression [41]. However, *FGG* (fibrinogen gamma chain) and *HBA1* (hemoglobin subunit alpha), significant contributors to response prediction at diagnosis, have not been comprehensively studied in CC cells. However, *FGG* dysregulation is known as a marker of radioresistance in head and neck cancer, which is also an HPV-related cancer [42]. For *HBA1*, the precise mechanism of non-responsiveness is still unknown. However, we hypothesized that this could be related to impairment of the oxygen transport mechanism. In clinical practice, anemia is well-known as a poor clinical prognostic indicator after CCRT [43,44]. However, in our cohort, we found no difference in anemic status. This implies that the unfavorable response after CCRT might be linked to issues with reduced oxygen transport proteins rather than anemia itself.

To further validate the credibility of the model, we separated both cohorts and performed analyses as training and test sets. While our observations revealed only modest positive identification metrics, they highlighted the model's outstanding negative prediction capabilities. Thus, the absence of these markers could play a pivotal role in augmenting the reliability and accuracy of confirming responsiveness in a patient cohort.

### 4.1 Limitations of the study

This study has some limitations. First, we could not quantify EVs from each method because of the limited amount after isolation, particularly in USEC. Therefore, we used plasma particles to indirectly measure the patient status. In addition, despite identifying the potential use of EV as a biomarker source to discriminate CCRT response status, the applicability could not be verified because of the relatively small number of patients. However, this study is the first step toward selecting a potential marker for future validation in more extensive clinical trials. Moreover, the significant proteins found at each time point could further assist in precise timing of biological fluid retrieval for validating each biomarker. Lastly, the limited timespan of follow-up duration hinders a thorough analysis of the correlation between EVs and the overall survival of LACC patients undergoing CCRT.

## 5 Conclusions

Our findings demonstrate dynamic biological responses after CCRT, revealing variations in EV isolation protocols, proteomic profiling, and temporal shifts in protein expression. Notably, the proteins involved in viral regulation and immune responses were significantly altered after treatment. Furthermore, our investigation identified potential biomarkers such as TFR1, FINC, FIBG, and HBA, which could discriminate between responders and non-responders to CCRT, offering valuable insights for prognostic assessment and therapeutic interventions. The validation of our model underscores its reliability in determining the responsiveness to CCRT, highlighting its potential clinical applicability. While our study presents promising avenues for biomarker discovery, further validation in larger clinical trials is warranted to translate these findings into clinical practice and enhance treatment outcomes for patients with LACC undergoing CCRT.

## Ethical statement and consent to participate

This study was approved by the Human Research Ethics Unit, all patients in the study provided written consent before participation (REC-63-465-4-2). as well as the informed consent was obtained from all the participants in accordance with the national legislation and the Declaration of Helsinki.

## Data availability statement

The MS/MS raw data and analysis files have been deposited in the ProteomeXchange Consortium (http://proteomecentral.proteomexchange.org) via the jPOST partner repository (https://jpostdb.org) with the data set identifier JPST002993 and PXD050754.

## Funding

The study was financially supported by the Research fund from the 10.13039/501100010804Faculty of Medicine, Prince of Songkla University (No. REC-63-465-4-2).

## CRediT authorship contribution statement

**K. Leetanaporn:** Writing – original draft, Visualization, Methodology, Investigation, Formal analysis, Data curation. **W. Chiangjong:** Resources, Methodology, Investigation, Data curation. **S. Roytrakul:** Resources, Methodology, Investigation, Data curation. **P. Molika:** Methodology, Investigation. **N. Janmunee:** Resources, Methodology. **T. Atjimakul:** Resources. **J. Hanprasertpong:** Writing – review & editing, Validation, Supervision, Project administration, Methodology, Formal analysis, Conceptualization. **R. Navakanitworakul:** Writing – review & editing, Validation, Supervision, Project administration, Methodology, Investigation, Funding acquisition, Conceptualization.

## Declaration of competing interest

The authors declare the following financial interests/personal relationships which may be considered as potential competing interests: Raphatphorn Navakanitworakul reports financial support was provided by Faculty of Medicine, 10.13039/501100004508Prince of Songkla University, Thailand. If there are other authors, they declare that they have no known competing financial interests or personal relationships that could have appeared to influence the work reported in this paper.
