# Supplementary material for: Enhancing outcome prediction of concurrent chemoradiation treatment in patients with locally advanced cervical cancer through plasma extracellular vesicle proteomics
Source: Heliyon. 2024 Aug 22;10(16):e36374. doi: 10.1016/j.heliyon.2024.e36374 (PMC11388600; doi:10.1016/j.heliyon.2024.e36374)

# CD9

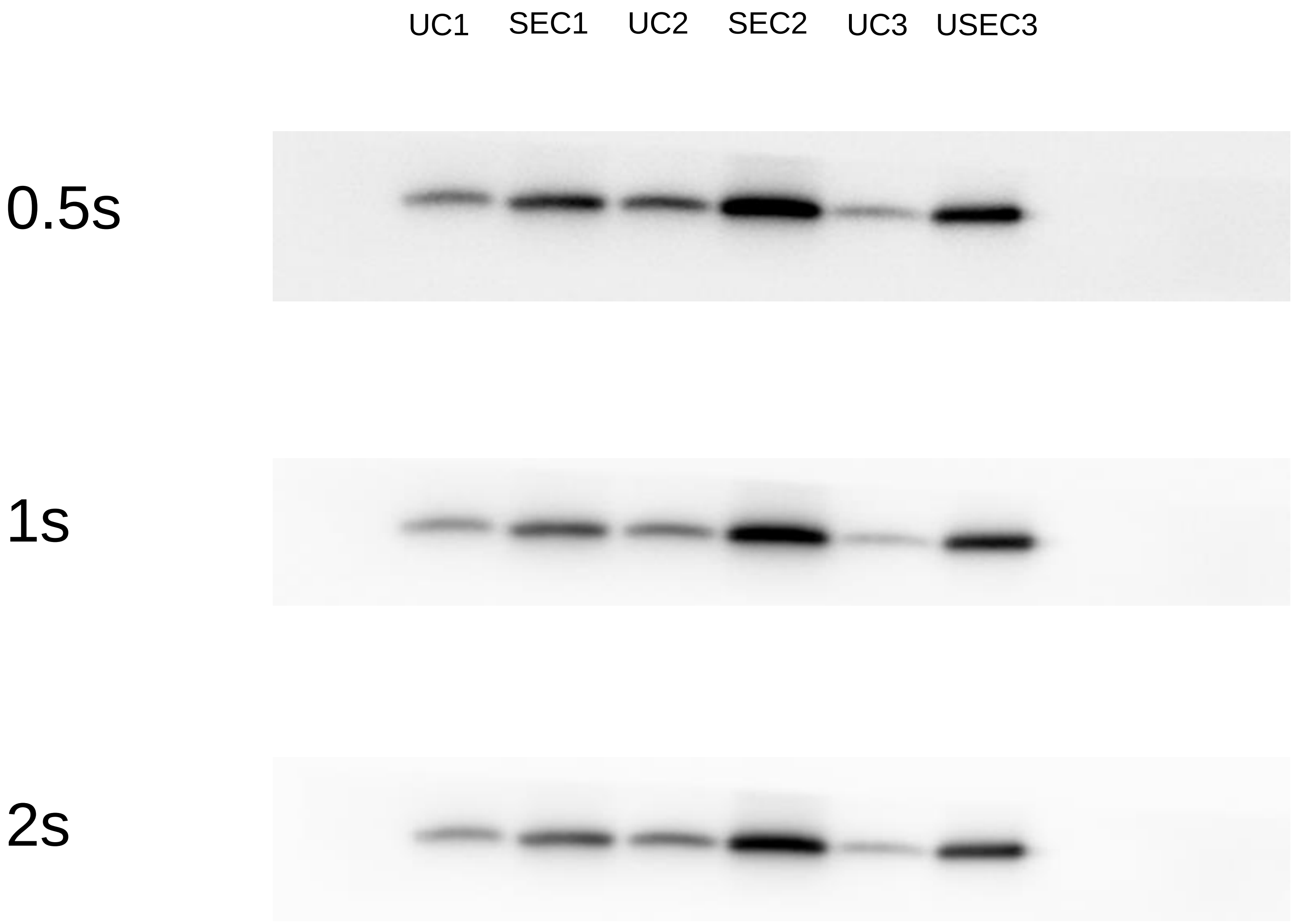

# CD63

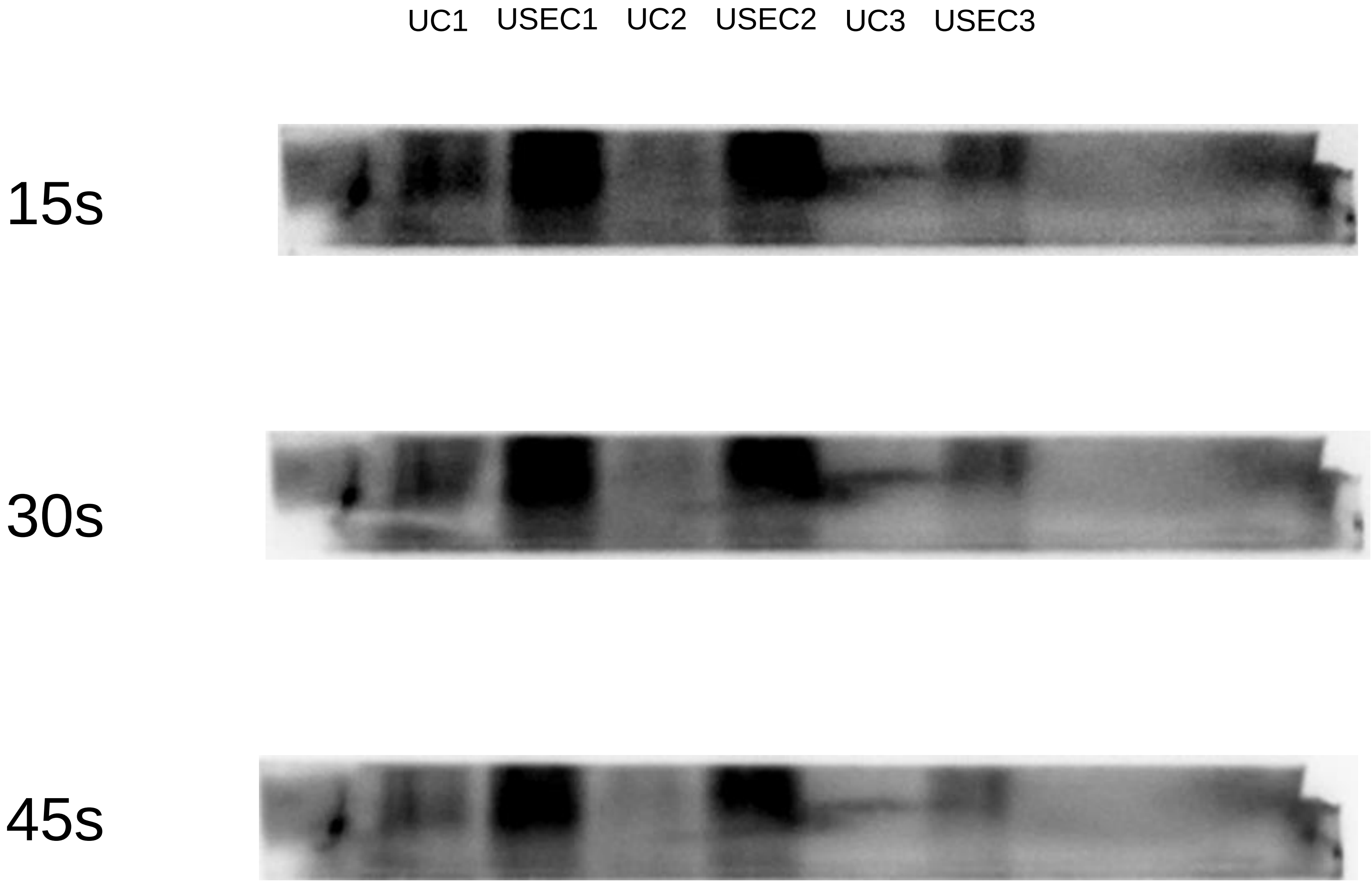

# Alb

UC1 USEC1 UC2 USEC2 UC3 USEC3

4s

8s

16s

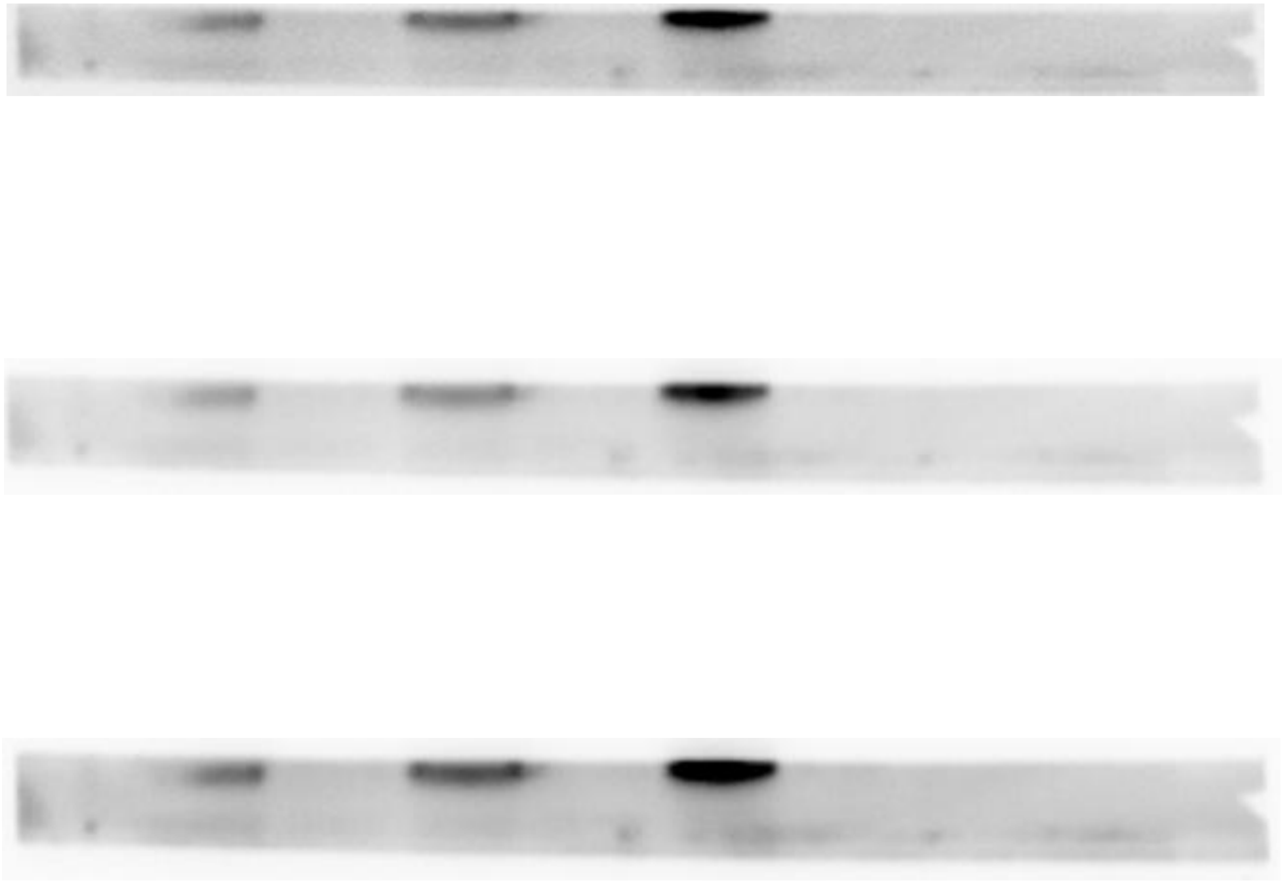

# Alix

UC1 USEC1 UC2 USEC2 UC3 USEC3

15s

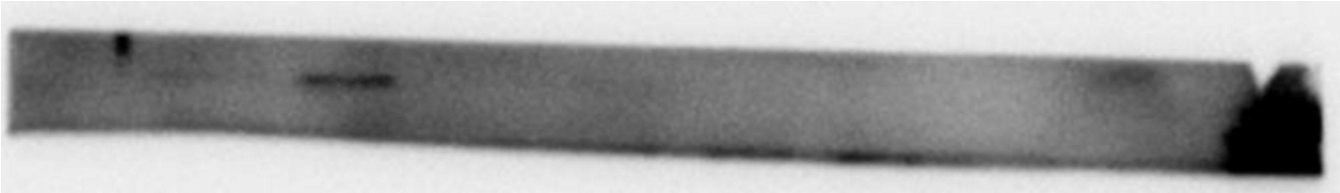

30s

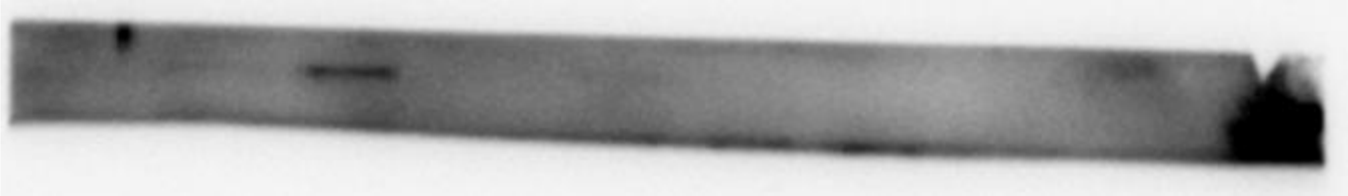

45s

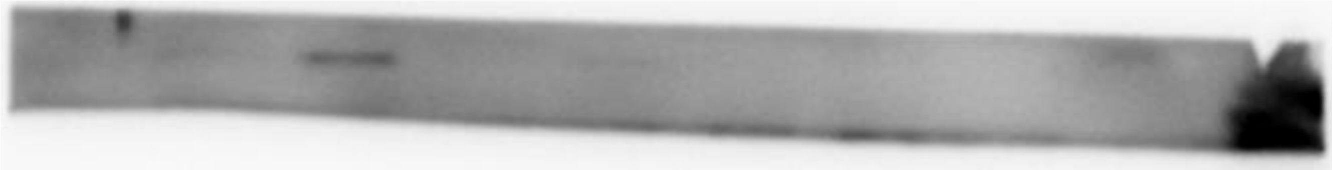

# CD81

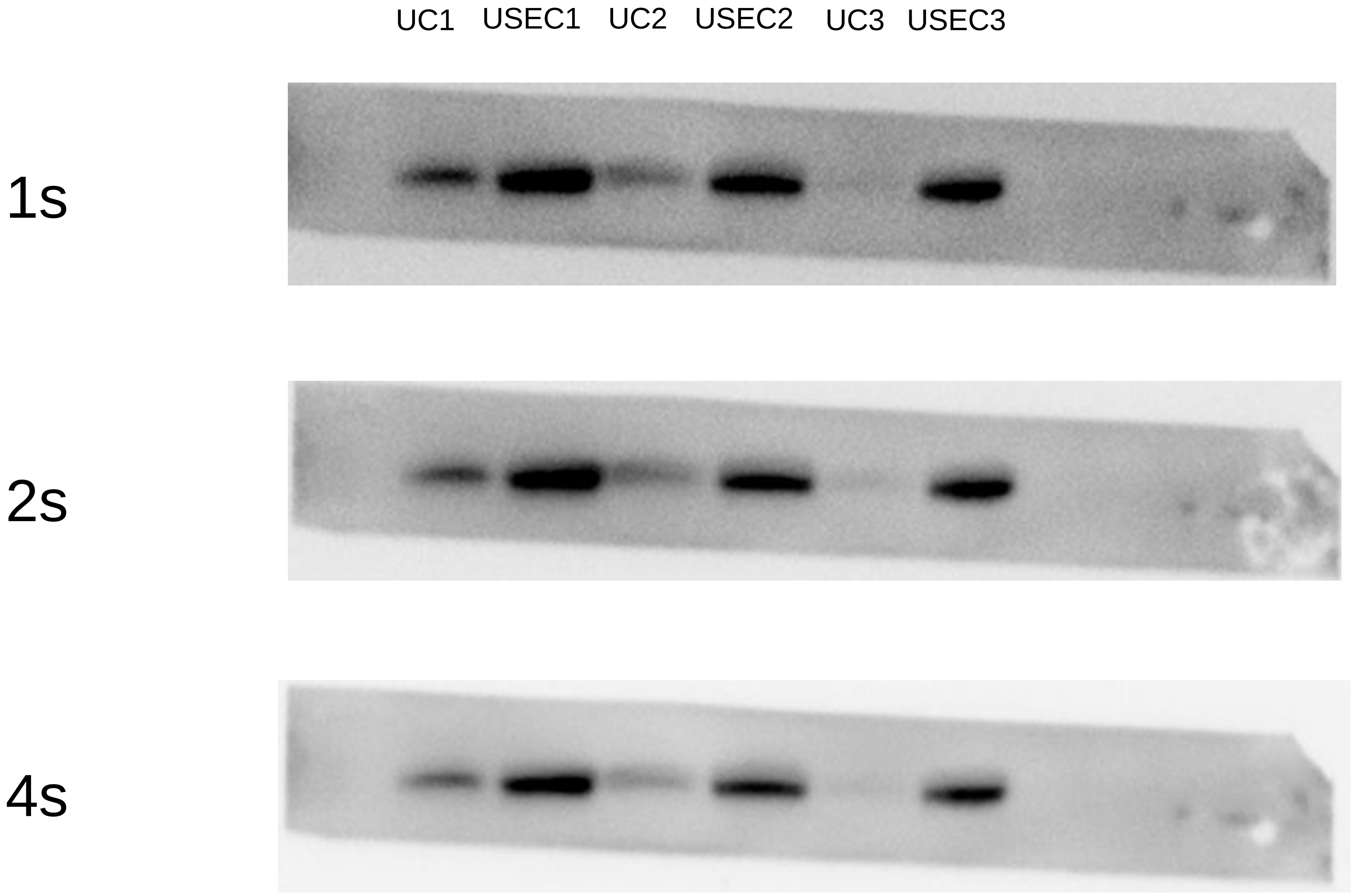

# Cyt C

UC1 USEC1 UC2 USEC2 UC3 USEC3

30s

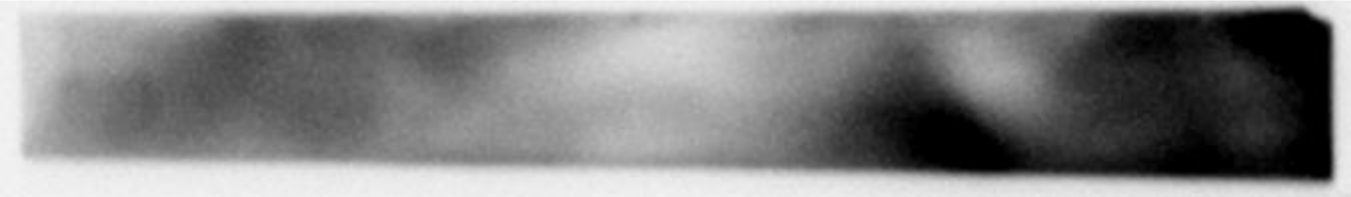

60s

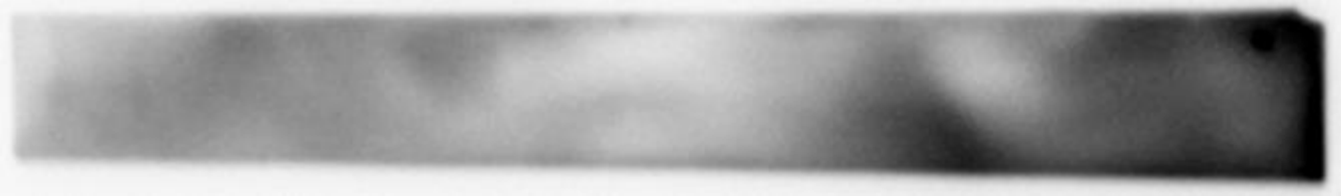

90s

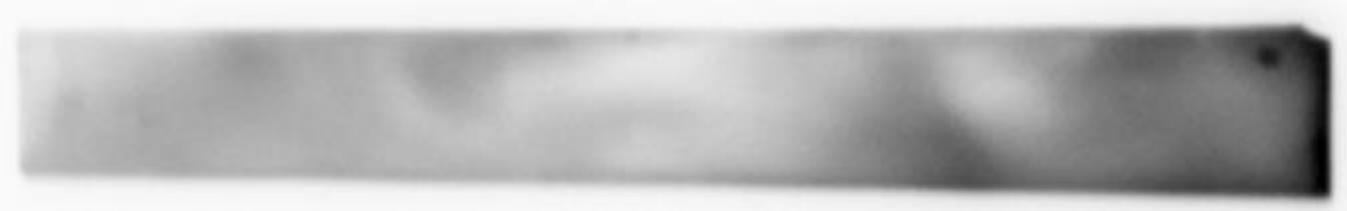

Supplement: Multimedia component 1 [file mmc1.pdf]
