# Supplementary figures and images for: Enhancing outcome prediction of concurrent chemoradiation treatment in patients with locally advanced cervical cancer through plasma extracellular vesicle proteomics
Source: Heliyon. 2024 Aug 22;10(16):e36374. doi: 10.1016/j.heliyon.2024.e36374 (PMC11388600; doi:10.1016/j.heliyon.2024.e36374)

**A**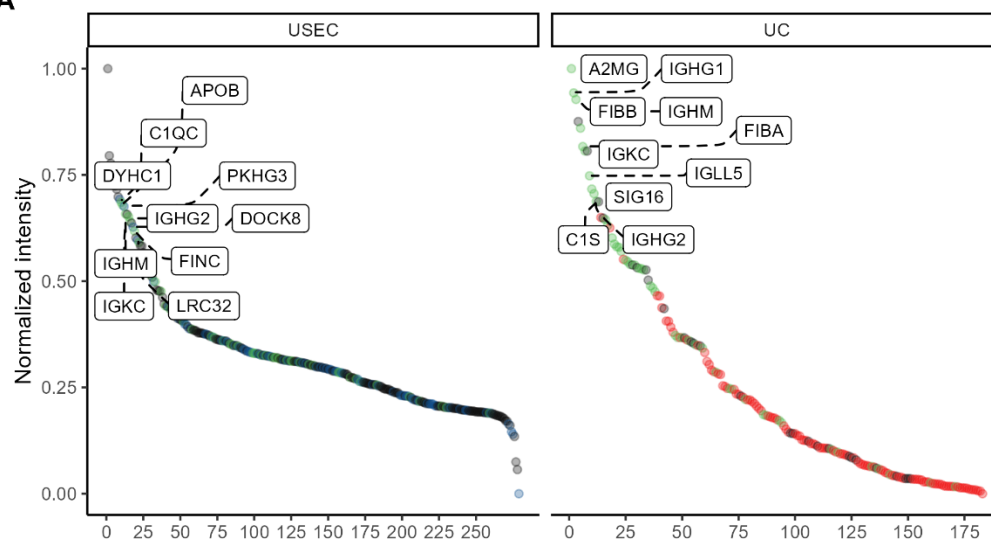**B**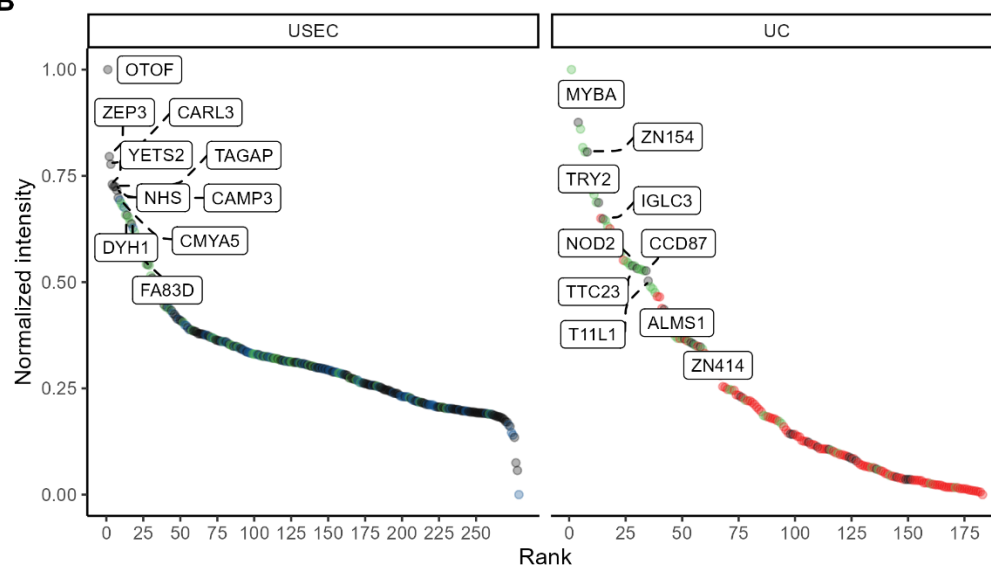

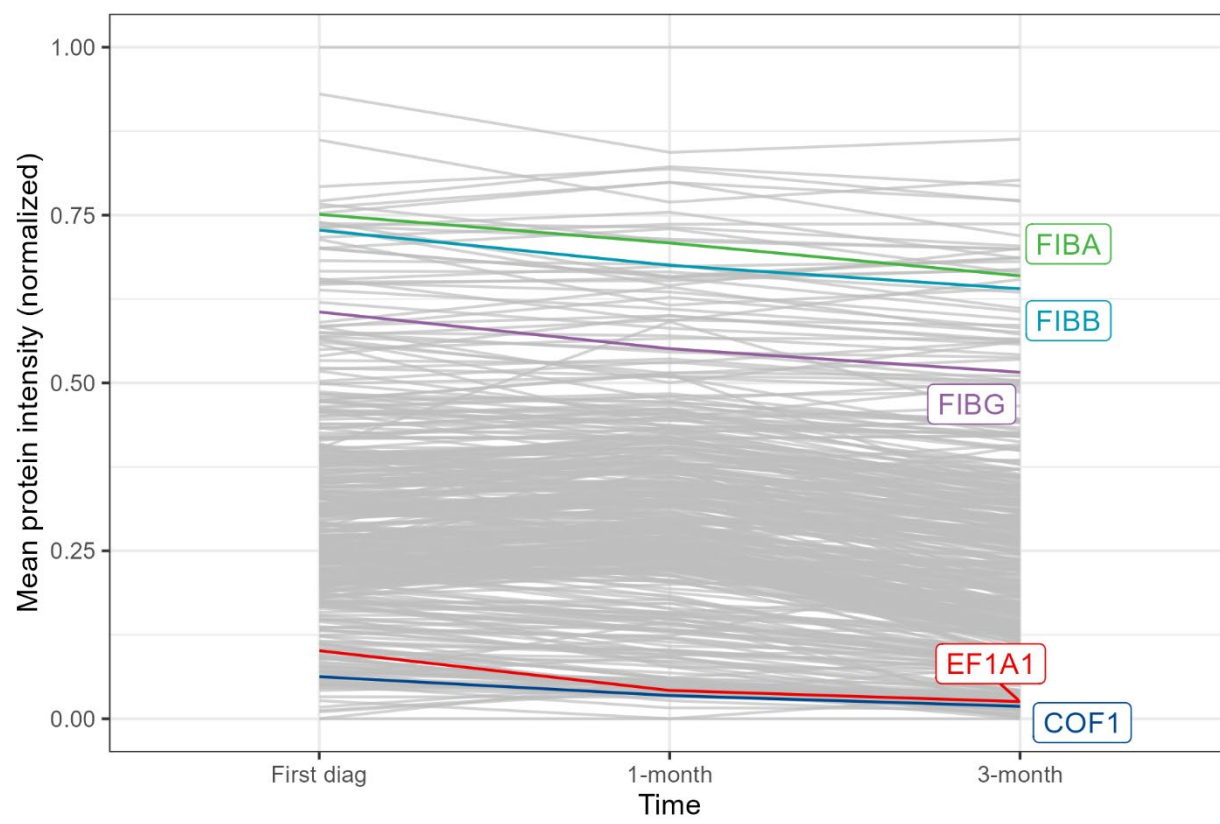

Supplementary figure 4

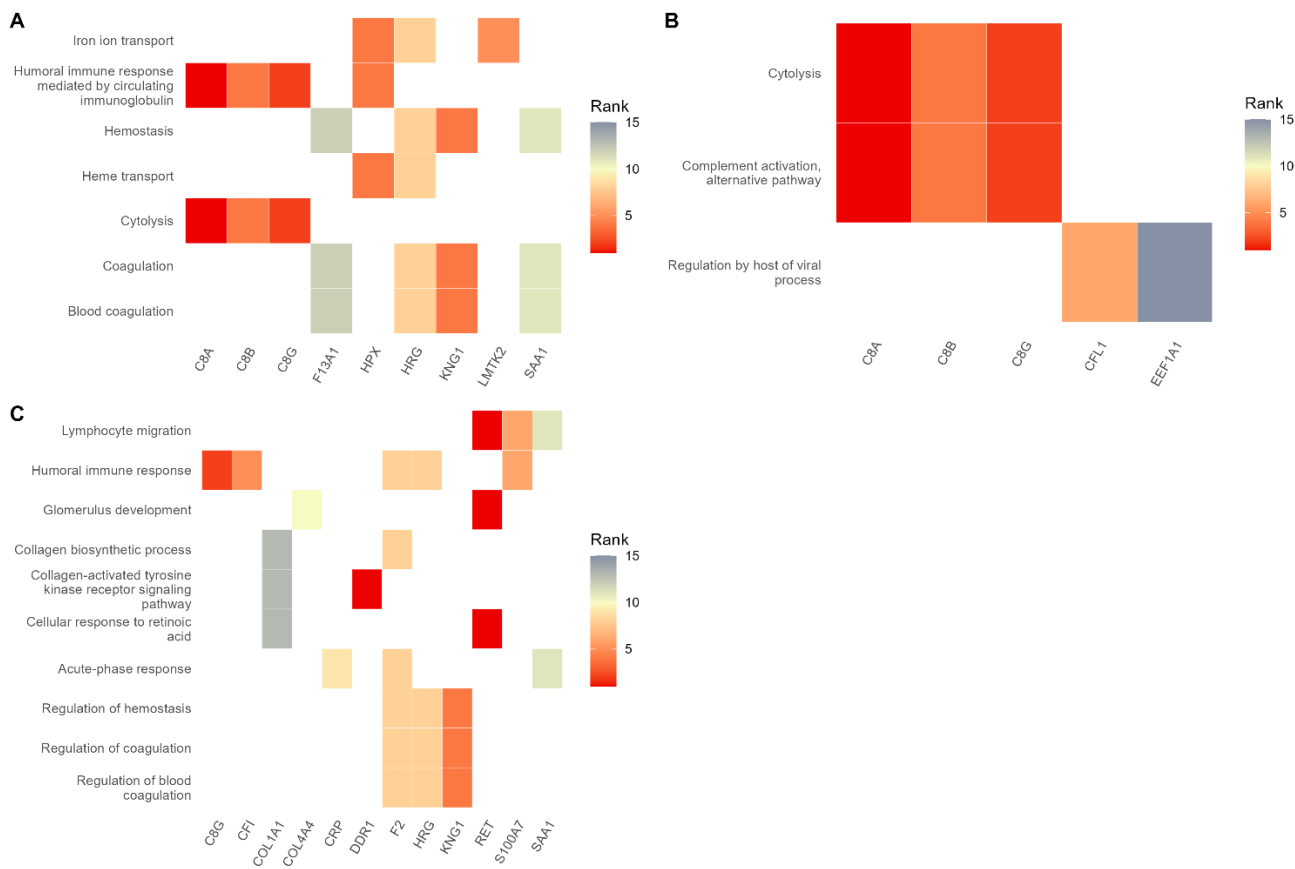

Supplementary figure 5

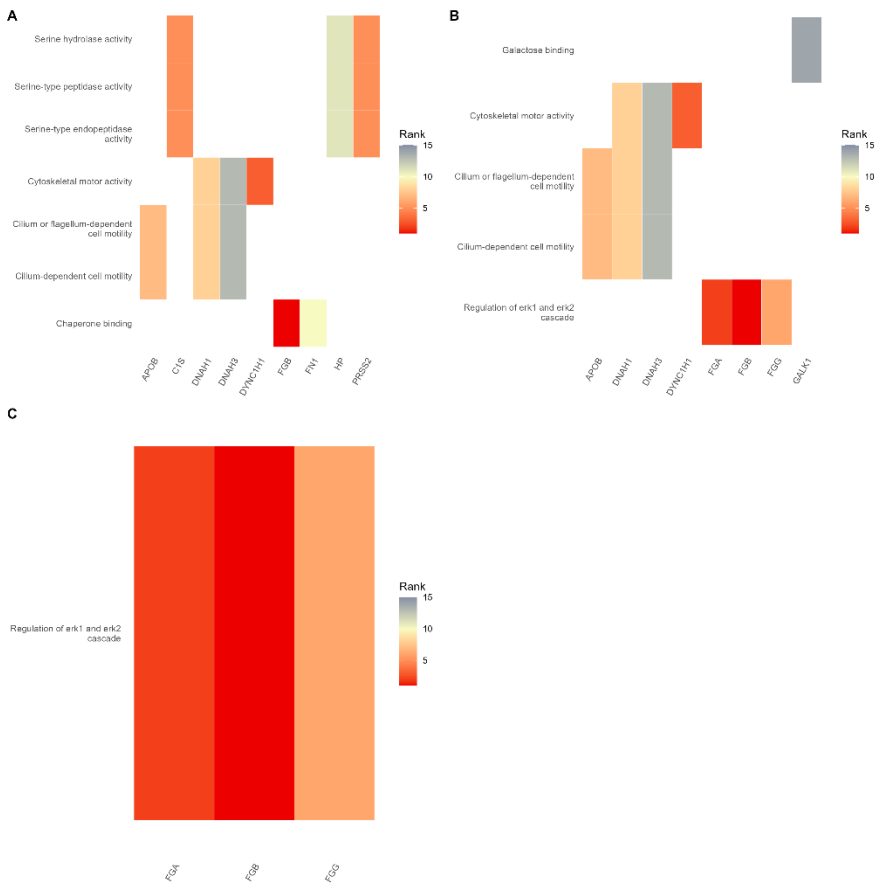

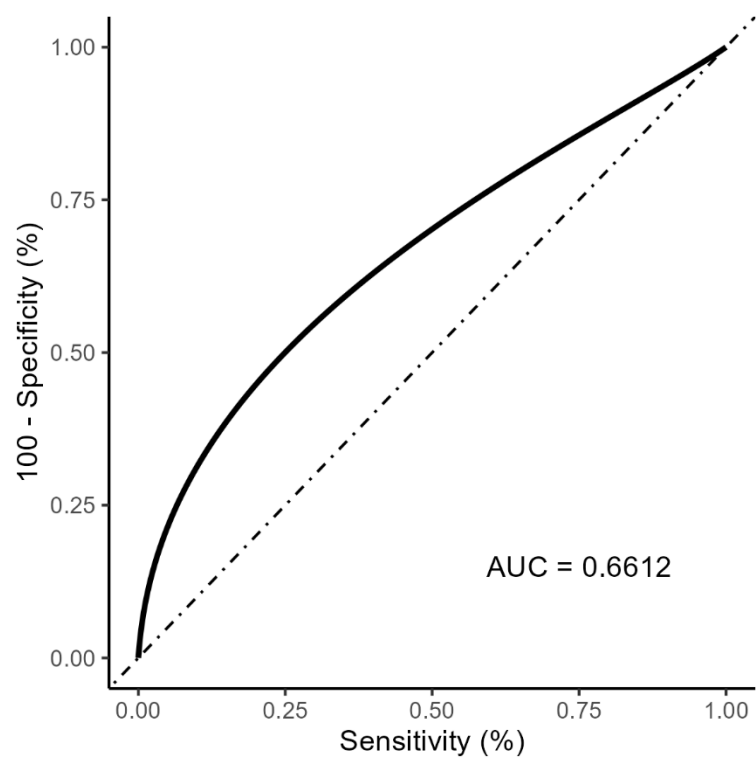

Supplementary figure 7

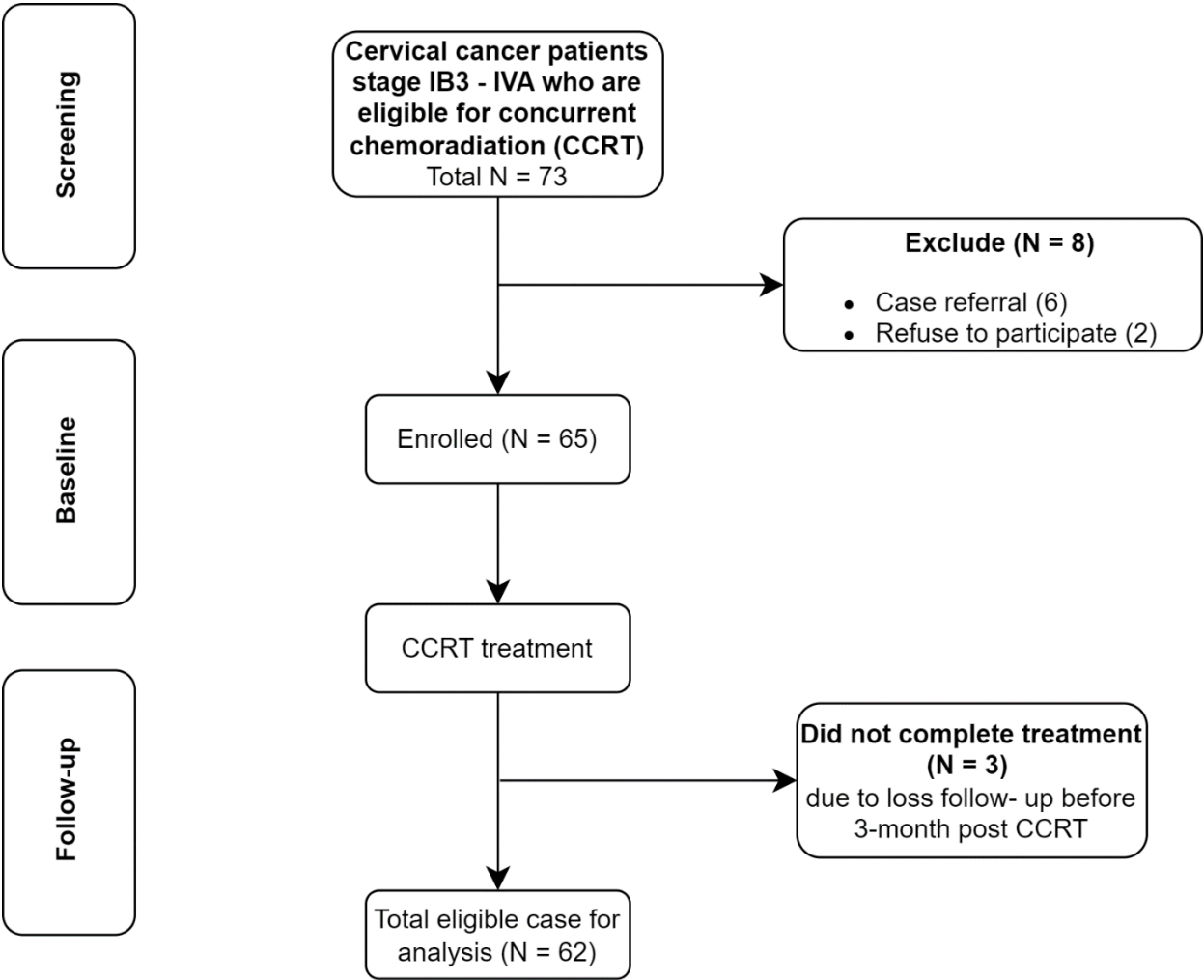

Supplement: Multimedia component 2 [file mmc2.pdf]
